# Treatment and outcome of a boy with lgG4-related hypophysitis caused by SARS-CoV-2 re-infection

**DOI:** 10.3389/fendo.2025.1663029

**Published:** 2025-11-19

**Authors:** Hanming Li, Iatlun Leong, Jianyu He

**Affiliations:** 1 Department of Pediatrics, The Fifth People’s Hospital of Foshan City, Foshan, Guangdong, China; 2 Department of General Surgery, University Hospital of Macau Special Administrative Region, Macau, Macao SAR, China

**Keywords:** IgG4-related disease, IgG4 related hypophysitis, SARS-CoV-2 infection, arginine vasopressin deficiency, central diabetes insipidus

## Abstract

**Rationale:**

SARS-CoV-2 infection can directly and indirectly affect the nervous system, including the hypothalamus and pituitary, and potentially cause IgG4-related hypophysitis.

**Patient concerns:**

A 4-year-old Chinese boy presented with arginine vasopressin deficiency (AVP-D, previously called ‘central diabetes insipidus’) and significant growth retardation. MRI indicated thickening of the pituitary stalk and alterations in the posterior pituitary.

**Diagnosis:**

The boy experienced polydipsia, polyuria, and enuresis 4 months after infection by SARS-CoV-2 and 2 months prior to presentation in June 2023. The diagnosis was IgG4-related hypophysitis, AVP-D, and growth hormone deficiency. Treatment with glucocorticoids and desmopressin led to significant resolution of symptoms and normalization of pituitary morphology. However, a second SARS-CoV-2 infection was followed by recurrence of polydipsia, polyuria, and thickening of the pituitary stalk. This recurrence led to a final diagnosis of IgG4-related hypophysitis caused by SARS-CoV-2 infection.

**Interventions:**

Glucocorticoids and desmopressin alleviated the AVP-D. Growth hormone and a diet and exercise plan were recommended to manage his short stature. We plan to conduct a functional assessment of the gonadal axis after he is 6 years old.

**Outcomes:**

After 18 months, the polydipsia and polyuria were controlled, and an MRI showed significant thinning of the pituitary stalk. This is the first reported case of lgG4-related hypophysitis in a Chinese boy infected with SARS-CoV-2.

**Lessons:**

We successfully controlled clinical symptoms, but further follow-up observations are needed to assess recovery. Although the role of SARS-CoV-2 infection in this patient’s condition is only suggestive, other reports have described a relationship between SARS-CoV-2 infection and lgG4-related hypophysitis.

## Graphical Abstract

Timeline.

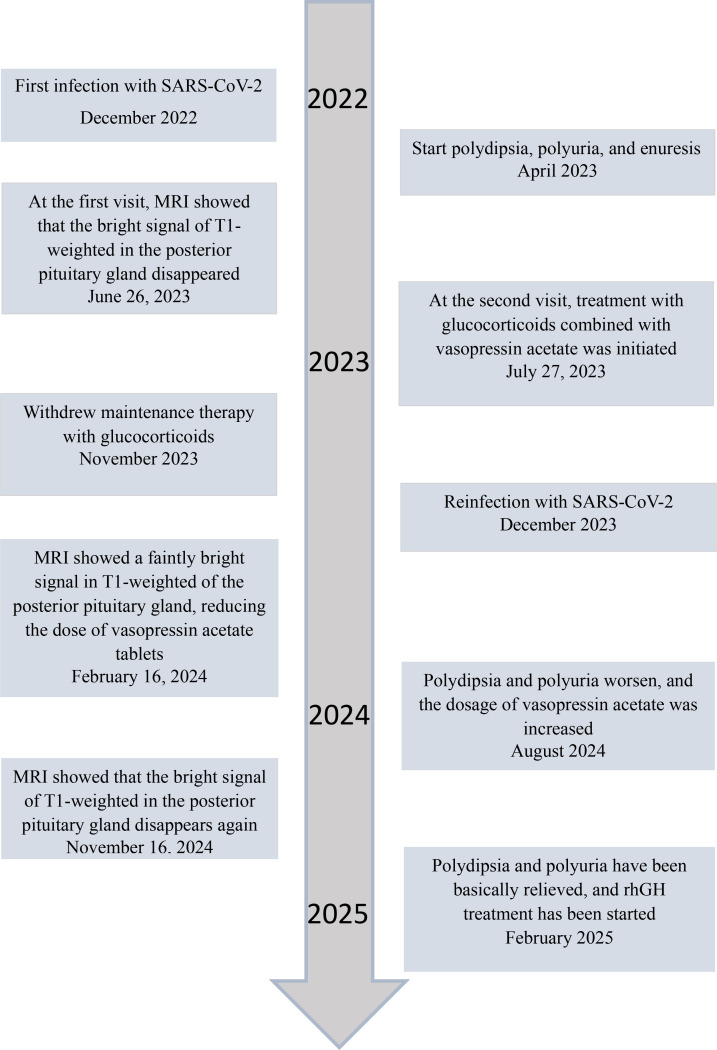

## 1 Introduction

IgG4-related disease (IgG4-RD) is a recently described immune-mediated chronic inflammatory disorder in which patients experience ‘mat-like’ fibrosis, infiltration of lymphocytes and IgG4+ plasma cells into various tissues, obliterated phlebitis, and eosinophilia (1). Although some patients are only affected in a single organ, most patients have lesions in multiple organs at the same time or in succession, and this disease can affect almost every part of the body. A significantly elevated serum level of IgG4 and lump-like lesions are the most common clinical manifestations, and the resulting fibrosis and persistent immune inflammatory response can cause compression and irreversible damage to affected organs and surrounding tissues, a condition that can lead to organ failure (2, 3). The diagnosis of IgG4-RD can be made by CT-PET imaging, flow cytometry with quantification of plasmablasts, and reverse passive latex agglutination that prevents negative results from the prozone phenomenon (4). Histopathologic confirmation is the gold standard for diagnosis.

Recent basic and translational research has identified the presence of different cellular subtypes and cytokines in the pathology of IgG4-RD and in the fibrosis of affected tissues (5). The formation of tertiary lymphoid tissues (TLTs) in affected organs is crucial, because T follicular helper (Tfh) 2 cells within TLTs promote the differentiation of IgG4+ B cells and contribute to lesion formation. The production of interleukins by these cells (IL-4 and IL-10) is an essential part of the pathogenesis (6). Cytotoxic T cells and M2 macrophages also contribute to inflammation and fibrosis. In 2019, the American College of Rheumatology/European League Against Rheumatism (ACR/EULAR) described the diagnostic criteria for IgG4-RD (7, 8).

Hypophysitis (enlargement of the pituitary gland) is a potential clinical manifestation of IgG4-RD. IgG4-related hypophysitis is an autoimmune disease characterized by pituitary dysfunction and hormone deficiencies in the anterior and/or posterior pituitary. These patients can also have diverse clinical manifestations in the nervous system that are related to the affected tissues and disease severity, often without specific symptoms or signs. The diagnostic criteria for IgG4-related hypophysitis (9, 10) include: (*i*) infiltration of pituitary tissues by mononuclear cells (especially lymphocytes and plasma cells), with more than 10 IgG4-positive plasma cells per high-power field; (*ii*) pituitary magnetic resonance imaging that shows a sellar mass or thickening of the pituitary stalk; (*iii*) alterations of other organs based on biopsy; (*iv*) serum IgG4 level above 1400 mg/L; and (*v*) a significant decrease of space-occupying effects and symptoms following glucocorticoid therapy.

Many studies of patients with coronavirus disease 2019 (COVID-19), caused by infection with the severe acute respiratory syndrome coronavirus 2 (SARS-CoV-2), can lead to immune system dysregulation and induce autoimmune diseases, but the mechanism is unclear (11). A multimodal meta-analysis used seed-based d mapping (SDM) software to compare COVID-19 patients with healthy controls (HCs) and identified changes of function and structure in the temporal lobe, orbofrontal cortex (OFC), and cerebellum, and that these changes were accompanied by functional or structural alterations in the insula and the limbic system and in gray matter volume (12). Structural and regional alterations on neuroimaging are common during the acute and chronic phases of COVID-19, particularly in the subcortical, prefrontal/frontal, and cortico-limbic brain areas, as well as the cerebrovascular/neurovascular system (13). A 2022 study of encephalitis patients with COVID-19 reported that SARS-CoV-2-associated or -mediated encephalitis was the most common manifestation (59.3%), and this was followed by autoimmune encephalitis (18.7%). Among all patients in this study, 66.7% were discharged (37.8% with improvement and 28.9% with full recovery), and 20.0% died (14). Although rare, COVID-19-associated hypophysitis was reported as a distinct condition that had similar prevalence among males and females, an average age of onset of about 35 years, and primary symptoms of headache, polyuria, and polydipsia (indicative of a deficiency of angiotensin and vasopressin) (15). In contrast, primary autoimmune hypophysitis is more common in females and the typical symptoms are headache and visual impairment (15).

Although hypophysitis is not common in patients with COVID-19, SARS-CoV-2 can function like a neuropathogen and cause high rates of disability and fatality, even when a patient has no signs of respiratory illness (14).

## 2 Case presentation

In June 2023, a 4-year-old Chinese boy first presented with his parents at the Department of Pediatrics at the Fifth People’s Hospital of Foshan City (Guangdong Province) due to polydipsia, polyuria, and nocturnal enuresis during the previous 2 months. His height was 94.8 cm (3 standard deviations [SDs] below average for boys (16)), his weight was 13.3 kg, and his BMI was 14.8 kg/m^2^.

He is the only child in the family and was born at full term by vaginal delivery. At birth, his length was 49 cm, weight was 3.0 kg, and head circumference was 43.0 cm. Since April 2023, his parents have noticed frequent symptoms of polydipsia, in that he consumed 3000 to 4000 mL of water every day and even awoke at night to drink water. He also had an increased urine output, and enuresis occurred 1 to 3 times per night. In addition to his delayed growth, he was also irritable and restless, experienced sleep disruptions and decreased sweating, and often refused to eat. However, there was no significant weight loss and his motor function and language development were normal. He did not receive a SARS-CoV-2 vaccination.

Six months before the initial presentation (December 2022), a nasal sample tested positive for SARS-CoV-2 in a nucleic acid test performed at home (17). At that time, his parents administered a Chinese herbal medicine. He experienced a fever for 2 days and he recovered quickly. His parents were in good health and there was no evidence of consanguineous marriage and no relevant family history.

### 2.1 Clinical findings

At the initial presentation (June 2023), the physical examination indicated small stature (see above), a normal facial expression, and a palpable irregular bony depression (2.0×2.5 cm^2^) at the occipitoparietal junction. There was no evidence of chest deformities, such as rib flare, or other physical abnormalities.

**Figure 1:**
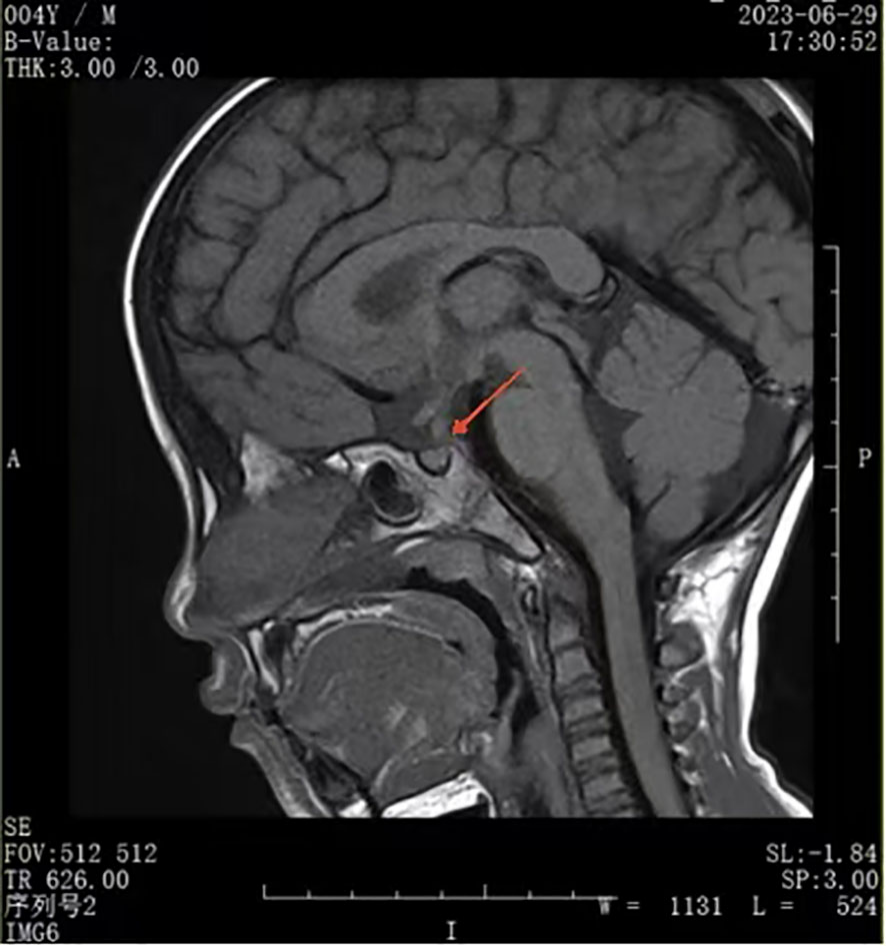
Nodular thickening of pituitary stalk.

A urinalysis demonstrated the specific gravity was 1.008 and the osmolality was 55.0 mOsm/L. A T1-weighted MRI of the pituitary gland indicated the absence of a bright signal in the posterior pituitary and nodular thickening in the upper part of the pituitary stalk, consistent with impaired function of the posterior pituitary. We considered the possibilities of pituitary inflammation and proliferative lesions (**Figure 1**). The symptoms and laboratory tests led to an initial diagnosis of arginine vasopressin deficiency (AVP-D, previously called ‘central diabetes insipidus’ (18)) and we planned to perform a water deprivation and vasopressin challenge test for confirmation.

However, his parents insisted on seeking treatment at a higher-level hospital, the Guangzhou Women and Children’s Medical Center (Guangdong). This other institution provided detailed laboratory results (**Table 1**) and results of a water-deprivation and vasopressin challenge test (**Table 2**). The examination at this other institution also reported no abnormalities in the thyroid, adrenal glands, urinary system, and in a cardiac Doppler ultrasound. However, their X-ray findings revealed abnormal changes in the bony structure at the occipitoparietal junction, suggesting a possible diagnosis of Langerhans cell histiocytosis. There were no abnormal bony changes in the chest, lumbar spine, pelvis, or right long bones.

**Table 1:** Detailed laboratory results.

Parameter	Results	Reference range
Urinary osmotic pressure	80.4mOsm/L	600-800mOsm/L
Plasma osmotic pressure	274mOsm/L	280-310mOsm/L
Triglyceride	1.54mmol/L	0.56-1.7 mmol/L
Total cholesterol	4.45mmol/L	3.1-5.7 mmol/L
Fasting blood glucose	5.01mmol/L	3.9-6.1 mmol/L
Fasting insulin	1.84 mU/L	3.0-25.0 mU/L
Uric acid	461.0μmol/L	149-416μmol/L
Prolactin	11.93ug/L	2.58-18.12ug/L
Cortisol	111.9nmol/L	140-630nmol/L
Free triiodothyronine	7.56pmol/L	3.5-6.5 pmol/L
Free tetraiodothyronine	20.67pmol/L	10.3-25.7pmol/L
Thyroid-Stimulating Hormone	1.22mIU/L	0.7-7.5mIU/L
Insulin-Like Growth Factor-1	93.3ng/mL	100–400 ng/mL
Testosterone	0.06 nmol/L	<0.24-0.97 nmol/L
Estradiol	72.38pmol/L	37–88 pmol/L
Follicle-stimulating hormone	1.44 IU/L	1.0-10.8 IU/L
Luteinizing hormone	0.10 IU/L	0.02-4.7 IU/L
Sodium	138.1mmol/l	135-145mmol/l
Potassium	4.24mmol/L	3.5-5.5mmol/L
Chlorine	101.7mmol/L	96.0-106.0mmol/L
Blood calcium	2.37mmol/L	2.25~2.75mmol/L
Blood phosphorus	1.79mmol/L	0.74~1.39mmol/L
25-hydroxyvitamin D	67.0nmol/L	20-100nmol/L
Blood pH	7.35	7.35~7.45

**Table 2:** Results of a water-deprivation and vasopressin challenge test.

Time	Urine output (ml)	Urinary osmotic pressure (mOsm/L)	Plasma osmotic pressure (mOsm/L)	Sodium (mmol/L)	Weight (kg)	Blood pressure (mmHg)	Desmopressin acetate	Others
7:00								water deprivation
7:14	160	74.6	274.0	136.3	13.2	116/72		
8:00	150	68.4			12.8	109/80		
9:00	290	82.3			12.6	88/62		
9:46	120	100.4	286.0	143.5	12.4	103/77		
11:00	120	169.9			12.3	101/76		
12:00	50	270.0			12.3	104/80		
12:45	50	286.2	319.0	145.0	12.1	106/73		End of water deprivation
12:55							0.02mg	vasopressin
13:19	35	323.8			12.1	108/73		
14:25	30	546.8						
8:00 next morning		74.2	283.0	142.5		102/72		

Based on their examination, clinicians at the Guangzhou Women and Children’s Medical Center diagnosed the child with AVP-D with Langerhans cell histiocytosis and prescribed oral tablets of desmopressin acetate (0.02 mg every 8 h). They also recommended a biopsy of the skull lesion. However, because the parents believed there was no significant improvement after treatment, they refused a biopsy and returned to our hospital.

### 2.2 Diagnostic assessment

At the second presentation at our hospital (July 27, 2023), his weight had decreased to 11.8 kg and he still had symptoms of polydipsia, polyuria and enuresis. He consumed 3000 to 4000 mL of water daily, his urine output was 8.5 to 12.0 mL/kg/h, and he had decreased appetite, lethargy, and sweating. Blood testing indicated his serum IgG4 level was 3630.0 mg/L (reference range: 130.0–1400.0 mg/L) and testing of his urine indicated significantly lower specific gravity and osmolality than in June 2023. A water deprivation test indicated low urinary osmotic pressure (<600 mOsm/L) and high plasma osmotic pressure (>300 mOsm/L) (19). A multidisciplinary consultation led to an initial diagnosis of suspected IgG4-related hypophysitis, AVP-D, and short stature.

### 2.3 Therapeutic intervention

Starting from August 2023, we gradually increased the dose of desmopressin from 0.05 mg each 12 h to 0.1 mg each 12 h (8 AM and 8 PM) and also initiated a low-dose glucocorticoid impact therapy consisting of intravenous methylprednisolone at 2 mg/kg/day. After one week, we changed the corticosteroid treatment to daily prednisone tablets (1.0 mg/kg each morning) and decreased the total dose of prednisone by 2.5 mg every week until reaching 2.5 mg per day. We continued the prednisone maintenance therapy for 3 months.

Soon after increasing the dose of desmopressin, the boy experienced significant decreases in polydipsia (750–1100 mL of water per day) and polyuria (1.0–3.2 mL/kg/h), his mood improved, and his nighttime sleep and appetite normalized. A physical examination revealed that the irregular bony depression at the occipitoparietal junction had decreased in size to 1.0×0.8 cm^2^.

**Figure 2:**
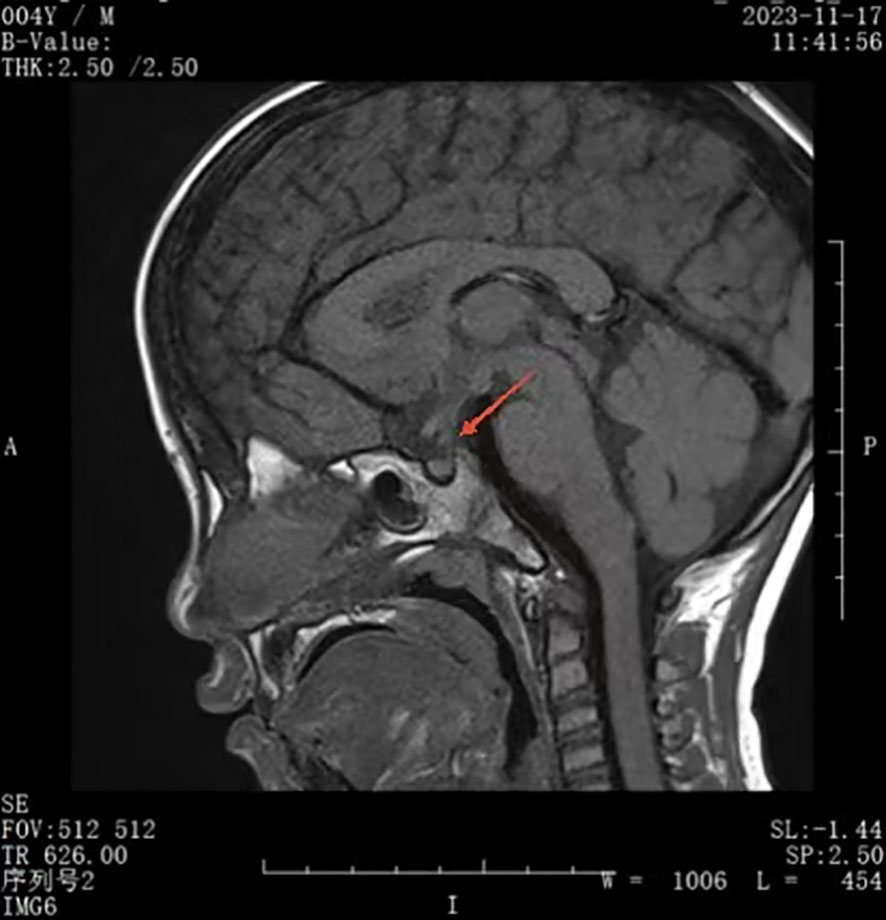
Reduction in thickening of pituitary stalk.

At a follow-up visit on November 17, 2023, a recheck of the serum IgG4 level showed a remarkable normalization (296.6 mg/L). Moreover, a pituitary MRI showed a marked reduction in thickening of the pituitary stalk, clearly defined borders, and no significant displacement (**Figure 2**). The T1-weighted image showed that the adenohypophysis had a normal shape and uniform signal intensity, and there was no bright signal in the posterior pituitary.

Based on these findings, we halted the low-dose prednisone therapy by the end of November 2023, but continued desmopressin treatment at 0.1 mg every 12 h. A skull X-ray showed complete resolution of the occipitoparietal junction of the vertex and occipital region.

In December 2023, the patient experienced fever symptoms again, but he quickly recovered without complications. His parents performed a home-based nucleic acid test for SARS-CoV-2 from a nasal sample, and the result was positive, but he had no abnormal symptoms during subsequent observations. His urine output, appetite, and sleep remained stable.

**Figure 3:**
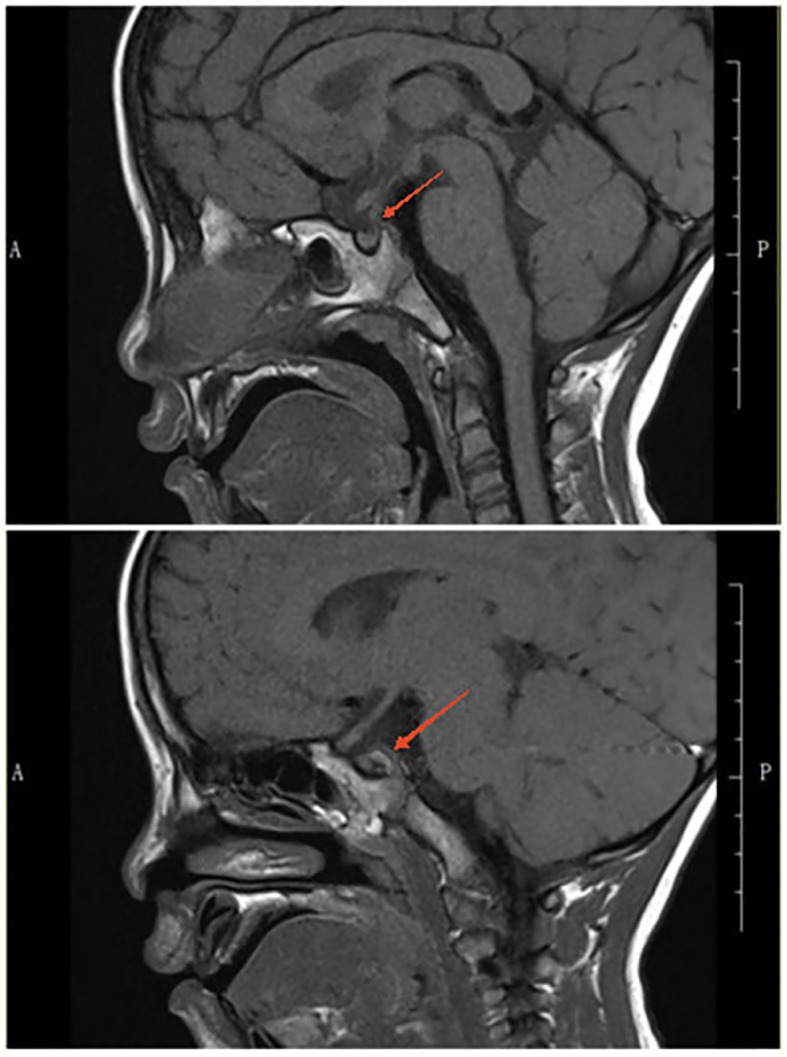
A faintly visible bright signal in the posterior pituitary.

At a follow-up visit on February 16, 2024, the serum IgG4 level was still normal (170.0 mg/L). The pituitary MRI showed mild thickening of the pituitary stalk, clear margins, and no obvious displacement; the adenohypophysis had normal morphology and a uniform signal; and the T1-weighted image showed a faintly visible bright signal in the posterior pituitary (**Figure 3**). Based on these findings, we began decreasing the dose of desmopressin. We maintained the 8 PM dose at 0.1 mg but decreased the 8 AM dose every 2 weeks (0.075 mg, 0.05 mg, and 0.033 mg) and finally ended the 8 AM dosing.

### 2.4 Follow-up and outcomes

After the second presentation, the entire treatment process was smooth and the boy’s daily water intake and urine output remained normal during the day. However, every time we decreased the 8 PM dose of desmopressin, he experienced recurrence of nighttime enuresis and polydipsia. Starting from August 2024, the boy experienced mild daytime polydipsia and polyuria, but the symptoms were significantly milder than when he first fell ill in April 2023. We believed this was related to his reinfection by SARS-CoV-2 in December 2023, and therefore increased the 8 AM dose of desmopressin to 0.033 mg.

**Figure 4:**
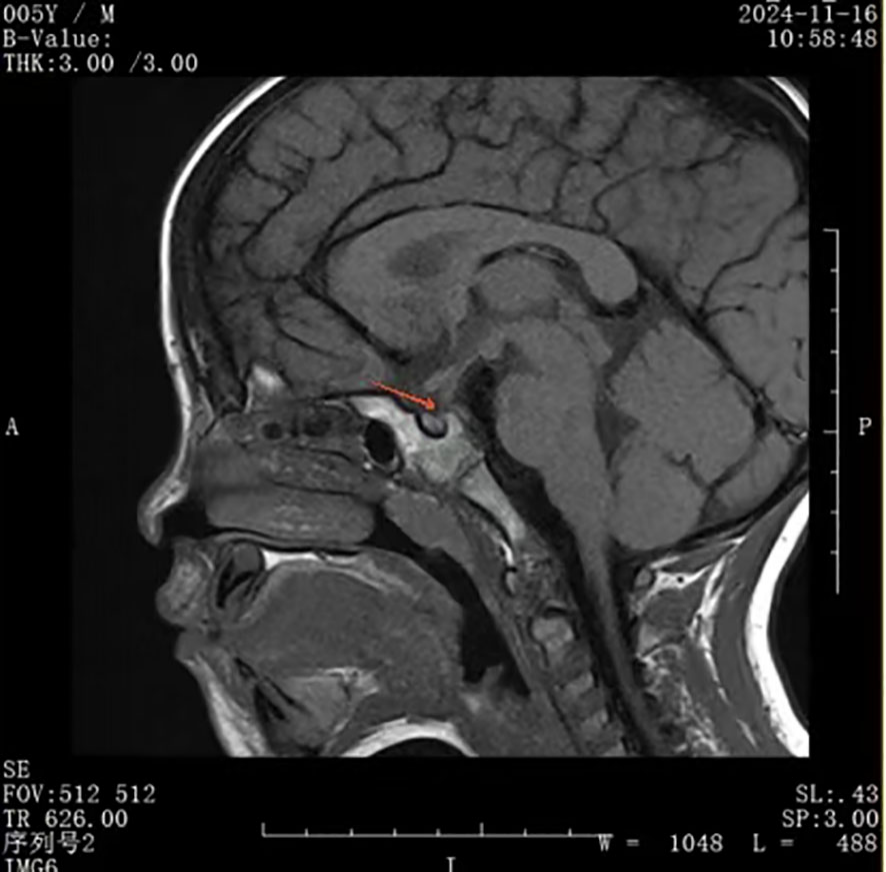
No bright signal in the posterior pituitary.

On November 16, 2024, his serum IgG4 level was slightly higher, but still in the normal range (577.0 mg/L) and a pituitary MRI showed no changes in the thickness of the pituitary stalk, normal adenohypophysis morphology with uniform signal intensity, and no bright signal in the posterior pituitary in the T1-weighted image (**Figure 4**). Despite recurrence of polydipsia and polyuria, we did not re-initiate glucocorticoid therapy because the serum IgG4 level was in the normal range and there was no significant change in pituitary morphology. However, we could not rule out the possibility of IgG4-related hypophysitis relapse due to glucocorticoid withdrawal.

In February 2025, the patient’s daytime symptoms of polydipsia and polyuria had largely resolved, and the urine specific gravity and osmolality returned to normal. We maintained the evening dose of desmopressin at 0.1 mg. After 18 months of this treatment, his weight increased to 16.0 kg and his height increased to 103.0 cm (3.2 SDs below average for boys (16)). Given the boy’s short stature, we conducted a bone age assessment test by X-ray of the hand and wrist, measured the levels of IGF-1, IGFBP-3, and performed a growth hormone stimulation test. The results indicated a deficiency of growth hormone (**Table 3**). Therefore, we initiated treatment with a short-acting growth hormone therapy (0.125 IU/kg/day by subcutaneous injection).

**Table 3:** Results of a growth hormone stimulation test.

Growth hormone stimulation test	Arginine	Levodopa
0 minutes	0.51μg/L	0.34μg/L
30 minutes	1.26μg/L	2.66μg/L
60 minutes	3.39μg/L	4.76μg/L
90 minutes	1.06μg/L	3.28μg/L

In May 2025 (3 months after initiation of growth hormone therapy), the patient’s height increased to 105.6 cm (2.8 SDs below average for boys (16)). Re-examination of the serum IgG4 level showed a slight increase to 1040.0 mg/L. We plan to perform a pituitary MRI after the IgG4 level decreases.

## 3 Discussion

The most common causes of a thickened pituitary stalk in children are germ cell tumors, Langerhans cell histiocytosis, and lymphocytic infundibulo-neurohypophysitis (20). A patient presenting with idiopathic pituitary stalk thickening and AVP-D should receive a dynamic pituitary function test, pituitary imaging by a specialist, a chest X-ray, abdominal ultrasonography, an optometry exam, and a skeletal survey for occult disease (21).

Our patient presented with symptoms typical of AVP-D (polydipsia and polyuria) and a water-deprivation-vasopressin test confirmed this diagnosis. As expected, his condition improved after administration of oral desmopressin (22). We also excluded the possibility of arginine vasopressin resistance (AVP-R, previously ‘nephrogenic diabetes insipidus’) caused by a loss-of-function mutation in the arginine vasopressin receptor 2 gene (*AVPR2*) (23). Therefore, we did not analyze the copeptin level (24, 25). However, we generally support the utilization of the outpatient copeptin stimulation test during the early stages of evaluation of polyuria-polydipsia syndrome in children (26). Autoimmune AVP-D should be considered, especially in the differential diagnosis of idiopathic AVP-R and when there are lesions of the sellar region (27).

We attributed the patient’s irregular bony defect at the occipitoparietal junction to calcium and phosphorus loss caused by AVP-D, although it was initially suspected to be a symptom of Langerhans cell histiocytosis. Following the control of AVP-D, the irregular bony defect resolved, and this was a crucial factor that excluded the previously suggested diagnosis of Langerhans cell histiocytosis (28).

During the course of severe COVID-19, a patient can experience acute and delayed effects on the pituitary that are related to the infection and/or treatment (29). One study concluded that positivity for anti-rabphilin-3A antibodies suggested that COVID-19-associated pituitary dysfunction was hypophysitis that involved an abnormal immune mechanism (30). Interestingly, pituitary stalk enlargement and positivity for anti-rabphilin-3A antibodies are also common in patients with SARS-CoV-2 vaccination-induced AVP-D (31). Our patient did not receive the SARS-CoV-2 vaccination, and there is evidence of a rapid onset of AVP-D after vaccination in some patients (32).

Our patient experienced two SARS-CoV-2 infections: December 2022 and December 2023. He developed symptoms of AVP-D in April 2023 (4 months after the first infection), and an MRI showed the disappearance of the bright T1-weighted signal in the posterior pituitary until June 2023. After treatment with glucocorticoids and oral desmopressin, his symptoms resolved. In February 2024 (2 months after the second infection), a follow-up MRI revealed a faintly visible bright T1-weighted signal in the posterior pituitary, indicating the recovery of posterior pituitary function.

However, the patient was reinfected with SARS-CoV-2 in December 2023, and he again experienced daytime polydipsia and polyuria in August 2024, although the symptoms were significantly milder than those in 2023. We therefore increased the 8 AM dose of oral desmopressin to 0.033 mg to control these symptoms. In November 2024, an MRI showed that the bright signal of the T1-weighted image of the posterior pituitary gland had disappeared again. Moreover, the boy’s serum IgG4 level was associated with the severity of symptoms.

Therefore, we believe that our patient developed lgG4-related hypophysitis due to SARS-CoV-2 infection, and this led to AVP-D and growth hormone deficiency. Regrettably, because the patient was tested for SARS-CoV-2 infection using a home-based test, we are unable to identify the specific viral strain. However, we emphasize there is no direct evidence indicating a causal relationship between SARS-CoV-2 infection and IgG4-related hypophysitis. More research is needed for confirmation.

A rapid response to glucocorticoids is a hallmark of IgG4-RD, and glucocorticoids and B-cell depletion are effective in most of these patients (33). We used a low-dose glucocorticoid impact therapy for the treatment of our patient to prevent the occurrence of glucocorticoid shock (34). Our subsequent clinical observations demonstrated the effectiveness and safety of this approach. The thickening of the pituitary stalk in our patient decreased significantly after the low-dose glucocorticoid impact therapy, and the polydipsia-polyuria symptoms also decreased. The recurrence of polydipsia-polyuria symptoms in August 2024 may be due to the second infection by SARS-CoV-2 in December 2023 or the failure of glucocorticoid maintenance therapy. This was a key focus during follow-up visits, because most clinical experience with IgG4-RD is from studies of adults.

A previous study proposed a preliminary distinction of the proliferative and fibrotic phenotypes of IgG4-RD based on clinical presentation, although IgG4 levels do not reflect relapse or long-term control (35). Induction treatment with a glucocorticoid is usually effective, but the tendency to relapse complicates the strategy for maintenance therapy. However, manifestations that indicate prominent tissue fibrosis on histological examination can be less responsive to glucocorticoid therapy than other types of IgG4-RDs (34). The maintenance of immunosuppressants, with or without a low-dose glucocorticoid, can be superior to withdrawal of combined glucocorticoid and immunosuppressant therapy for preventing relapse in patients with long-term IgG4-RD (36). Current research is exploring several novel approaches, including depletion of B-cells, targeted immunomodulation of B cells, inhibition of Bruton’s tyrosine kinase, disruption of co-stimulation pathways, blockade of SLAMF7 (a self-ligand) or its signaling cytokines (BAFF, IL-4, or IL-6), and inhibition of JAK-STAT signaling (6). These emerging strategies hold the promise of improving the outcomes of patients with IgG4-RD.

Even though our patient’s clinical systems were significantly affected, there was apparently no overall or long-term loss of biochemical control in the pituitary. His growth delay also responded well to treatment with growth hormone. We are planning a follow-up MRI to assess recovery of the posterior pituitary and will also closely monitor his gonadal function, which could be impaired by lgG4-related hypophysitis and is therefore of great importance.

## Data Availability

The original contributions presented in the study are included in the article/supplementary material. Further inquiries can be directed to the corresponding author.
